# Validity and Reliability of the Persian Version of Parkinson's Disease Sleep Scale-2

**DOI:** 10.1155/2021/2015123

**Published:** 2021-12-20

**Authors:** Mohammad Taghi Joghataei, Seyed-Mohammad Fereshtehnejad, Maryam Mehdizadeh, Sepideh Goudarzi, Sayed Amir Hasan Habibi, Mahsa Meimandi, Arian Dehmiyani, Ghorban Taghizadeh

**Affiliations:** ^1^Department of Neurosciences, Faculty of Advanced Technologies in Medicine, Iran University of Medical Sciences, Tehran, Iran; ^2^Cellular and Molecular Research Center, Iran University of Medical Sciences, Tehran, Iran; ^3^Division of Clinical Geriatrics, Department of Neurobiology, Care Sciences and Society (NVS), Karolinska Institutet, Stockholm, Sweden; ^4^Division of Neurology, Faculty of Medicine, University of Ottawa, Ottawa, Ontario, Canada; ^5^Department of Pharmacology and Toxicology, Faculty of Pharmacy, Tehran University of Medical Science, Tehran, Iran; ^6^Department of Neurology, Rasoul Akram Hospital, Iran University of Medical Science, Tehran, Iran; ^7^Rehabilitation Research Center, Department of Occupational Therapy, School of Rehabilitation Sciences, Iran University of Medical Science, Tehran, Iran

## Abstract

**Objective:**

Sleep problems are nonmotor symptoms in Parkinson's disease that should be carefully evaluated for better management and treatment. Parkinson's Disease Sleep Scale (PDSS-2) is one of the most reliable tools for measuring sleep difficulties in people with Parkinson's disease. This study investigated the psychometric properties of the Persian version of PDSS-2.

**Methods:**

Four hundred and fifty-six people with Parkinson's disease with a mean age ±standard deviation of 60.7 ± 11.3 years were engaged in this study. Acceptability was assessed by floor and ceiling effects. Dimensionality was measured by exploratory factor analysis. The convergent validity of PDSS-2 with the Hospital Anxiety and Depression Scale (HADS) was assessed. Internal consistency and test-retest reliability were assessed with Cronbach's alpha and intraclass correlation coefficient (ICC), respectively.

**Results:**

No noticeable ceiling and floor effect was detected. The dimensionality analysis showed three factors. A high correlation was obtained between PDSS-2 and HADS (anxiety subscale). Excellent internal consistency with *α* = 0.94, and good test-retest reliability with ICC = 0.89 were obtained.

**Conclusion:**

This study showed that the Persian version of Parkinson's Disease Sleep Scale has acceptable validity and reliability for measuring sleep disturbances in people with Parkinson's disease.

## 1. Introduction

Symptoms of Parkinson's disease (PD) falls into two categories: motor (bradykinesia, rigidity, and tremor) and nonmotor (cognitive problems, pain, sleep difficulties, dysautonomia, and hyposmia) [1, 2]. Sleep disorders are a common, crucial, and challenging problem among the nonmotor symptoms of PD which is present in about 80% of the people with Parkinson's disease, despite only 30% of them report these problems [3, 4]. Sleep disorders in people with PD include nocturnal akinesia with or without early morning dystonia, insomnia, sleep fragmentation with increased periods of wakefulness during the night, rapid eye movement sleep behavior disorder (RBD), restless legs syndrome (RLS), hallucinations, other neuropsychiatric disturbances, sleep apnea syndromes, and nocturia [2, 5, 6].

Because of high prevalence of sleep disorders and nocturnal problems in patients with Parkinson's disease, which may occur in both early and advanced stages of PD [7], there is a need for accurate tools to precisely measure sleep disorders in these patients. The primary test for assessing sleep problems is video-polysomnography (PSG), which requires special equipment and expertise to use, and cannot always be easily accessible [8]. For this purpose, questionnaires and interview-based instruments have been developed to assess sleep and nocturnal problems, including Parkinson's Disease Sleep Scale (PDSS), Pittsburgh Sleep Quality Index (PSQI), Scales for Outcomes in Parkinson's Disease-Sleep scale (SCOPA-Sleep), Epworth Sleepiness Scale (ESS), Inappropriate Sleep Composite Score (ISCS), and Stanford Sleepiness Scale (SSS) [9].

The PDSS is one of the most widely used and specialized tools in Parkinson's population, and a revised version of it was published in 2010 [4]. Recently, a new version of PDSS (PDSS-2) has been published, which emphasizes nocturnal symptoms such as sleep apnea and restless leg syndrome [4]. PDSS-2 has been validated in several languages, and acceptable psychometric properties have been reported for it. However, so far, this scale has not been translated and adapted for Iranian patients with Parkinson's disease [8, 10–13]. Therefore, this study aimed to evaluate the validity and reliability of the Persian version of PDSS-2.

## 2. Methods

### 2.1. Participants

In this study, 456 people (277 men) with Parkinson's disease (mean age ± standard deviation of 60.7 ± 11.3 years) who were referred to a movement disorders clinic in Tehran, Iran, were recruited through a nonrandom convenient sampling. Inclusion criteria were the diagnosis of idiopathic Parkinson's disease based on the UK Brain Bank criteria [14], ability to read and write in Persian language, lack of significant cognitive problems (Mini-Mental Status Examination > 24) [15], and not taking medications that can alter sleep behavior (i.e., benzodiazepines, antiepileptics, antipsychotics, antihistamines, and melatonin).

All individuals signed an informed consent upon recruitment. The Student Research Committee of Iran University of Medical Sciences approved the study protocol.

### 2.2. Data Collection

Demographic characteristics including age, sex, illness duration, dose of anti-Parkinson medications, and medical history were recorded. Subjects then completed the PDSS-2 and Hospital Anxiety and Depression Scale (HADS). To prevent any change in sleep habits, subjects were reevaluated 1–3 days later for retesting. All assessments were done 1 h after intaking anti-Parkinsonian drugs (on-state).

Parkinson's Disease Sleep Scale-2 (PDSS-2): there are 15 questions on various sleep disturbances in this questionnaire. Scoring of each question ranges from 0 (never) to 4 (very frequent), with the sum ranging from 0 to 60. Three main categories of sleep disturbances are defined in this questionnaire: (1) PD-specific symptoms (confusion, muscle cramps, pain, immobility, hallucinations, snoring, and pain), (2) motor symptoms (dystonia, akinesia, tremor during waking period at night, restless behavior, and periodic limb movements), and (3) sleep-specific disturbances (sleep maintenance problems, unrestored sleep in the morning, insomnia, and the subjective assessment of sleep quality) [8, 11].

#### 2.2.1. Hospital Anxiety and Depression Scale (HADS)

The HADS includes14 items and consists of two subscales: anxiety and depression. Each item is scored on a four-point scale. The higher score in anxiety and depression subscales indicates higher level of anxiety and depression [16]. As previous studies suggested, we utilized the anxiety subscale for the relationship analysis.

### 2.3. Translation

Translation into Persian was done following obtaining permission from the developer. The process followed international standards of translation [17]. The original version was primarily translated into Persian, which was discussed by a panel of experts including neurologists and healthcare specialists to check equivalence to the original version and correct language. In the second step, a backward translation was done by other expert translators. Both of the Persian and English versions were reexamined by therapists and patients, and if needed, corrections were made. The English version was then sent to the original authors of PDSS-2 to obtain final approval.

### 2.4. Statistical Analysis

Demographic characteristics (frequency, mean, and standard deviation) and PDSS-2 score were reported using descriptive statistics.

The acceptable ceiling and floor effect was calculated as less than 15% of participants with the maximum or minimum score and skewness of −1 to +1 [18].

In order to investigate the dimensionality, exploratory factor analysis was performed using varimax rotation (Kaiser–Meyer–Olkin (KMO)), and values between 0.8 and 1 indicated adequacy of sampling [19].

Convergent validity was determined by calculating the Spearman correlation coefficient (*ρ*) between the total score of PDSS-2 and HADS (anxiety subscale). The *ρ* values > 0.60 show a high correlation [20].

Internal consistency of PDSS-2 was calculated using Cronbach's alpha, in which its value greater than 0.80 indicates high internal consistency. To investigate the relationship between individual items, the interitem correlation was calculated, and value above 0.20 was considered acceptable [21].

The intraclass correlation coefficient (ICC) with a 95% confidence interval (two-way mixed model with consistency type) was calculated to assess the test-retest reliability. ICC above 0.70 indicates excellent reliability [22]. The standard error of measurement (SEM) was also calculated by (${\text{SEM}=\text{SD}}_{\text{pooled}}\sqrt{1-\text{ICC}}$), and SEM less than 10% of the total score was considered acceptable [23].

All data analyses were done with SPSS version 21.0 (SPSS Inc., Chicago, IL) with *P* value of significance set to < 0.05 [24].

## 3. Results

The main demographic characteristics of individuals with PD are given in Table 1. The total score of PDSS-2 was 21.3 ± 15 (ranging from 0 to 60). An acceptable ceiling and floor effect (0%) was obtained (skewness = 0.49).

In factor analysis, 15 items were assigned in three factors; items 4, 5, 8, 9, 10, 11, 12, 13, and 14 were in the first factor (PD motor symptoms at night), items 6, 7, and 15 were in the second factor (PD symptoms at night), and items 1, 2, and 3 were in the third factor (sleep disturbances), with total variance = 53.84 and KMO = 0.93 (Table 2).

Regarding the convergent validity analysis, a high correlation was obtained between PDSS-2 and HADS (anxiety subscale), │*ρ* = 0.72│.

Cronbach's alpha for internal consistency was 0.94, and the correlation between items ranged from 0.21 to 0.84 (Table 3). At test-retest reliability, the ICC value for the total score of PDSS-2 was 0.89 (95% CI = 0.86–0.90). Also, the SEM for this parameter was 6.66 (SD_pooled_ = 17.82).

## 4. Discussion

This study aimed to evaluate, for the first time, the validity and reliability of the Persian version of PDSS-2. The results showed that this scale has acceptable structural validity, internal consistency, and test-retest reliability in Iranian people with Parkinson's disease. Thus, PDSS-2 can be reliably used in this population to identify sleep-related problems associated with Parkinson's disease and to specify patients who need more specialized assessments such as polysomnography.

Our analysis showed that the total score of the Persian version of PDSS-2 did not have a noticeable ceiling and floor effect, which was consistent with the previous studies. In the study of Kovács et al., a slight ceiling effect was observed for all domains, and in the study of Martinez-Martin et al., a small ceiling effect was seen for item 8 (get up at night to pass urine) [11, 12].

In factor analysis, the items of the Persian version of PDSS-2 were divided into three factors (factor 1: “motor problems at night,” factor 2: “PD symptoms at night,” factor 3: “disturbed sleep”). In previous studies, the number of factors for this scale has been reported between 1 and 5 (e.g., 4 factors for Spanish version [12], 5 factors for Italian version [10], 1 factor for original version, and 3 factors for Hungarian version [11]). This different number of factors in different versions reflects the heterogeneity and culture-related nature of sleep-related disturbances [9–12, 25].

According to the results of convergent validity, a high correlation was found between the total score of PDSS-2 and HADS (anxiety subscale), which is in line with previous studies. According to these research studies, the presence of anxiety may affect sleep quality, and also, sleep disturbances may cause anxiety, indicating a two-way relationship between these two highly prevalent nonmotor symptoms of Parkinson's disease [1, 3, 26, 27].

In the study of internal consistency, consistent with the results of former studies [4, 10, 12], excellent Cronbach's alpha was obtained. This demonstrates the coherence within the 15 items of the PDSS-2 scale [28].

The test-retest reliability analysis indicated the reproducibility of the Persian version of PDSS-2, which is in line with previous studies in different populations [4, 10, 12, 29]. In examining the psychometric properties of a clinical instrument, it is essential to assess the stability of repetitive responses or the stability of the responses over time. The test-retest reliability can be influenced by factors such as physical or mental changes in the respondent and/or evaluators or receiving new medication. To assure that variations in the sleep patterns of each individual would not significantly affect the results [30], we considered a three-day interval between test and retest as suggested previously [4].

SEM is a variable used for the responsive stability, which was less than 10% of the total score in our study, meaning that our SEM is accurate enough. We have therefore concluded that the responsiveness in our assessments and interventions was adequate [4, 10–12].

ne of the limitations of this study was the lack of assessing convergent validity of PDSS-2 using specific sleep scales due to the unavailability of reliable and valid Persian versions of such scales in PD patients. Another one was that participants were recruited from a single center and not multicenters. The exclusion of patients with cognitive deficit also limits the generalization of our findings as evidence has shown that early cognitive impairment accompanies sleep disorders (in particular RBD) in PD [31]. It is therefore suggested that these limitations be considered in future studies in addition to examining the responsiveness of the PDSS-2.

## 5. Conclusion

Overall, the results of this study confirmed that the Persian version of PDSS-2 has appropriate validity and reliability for assessing sleep-related disorders in Iranian patients with Parkinson's disease. This scale can also determine the need for sleep interventions and referral to sleep clinics for specialized treatment of sleep disturbances.

## Figures and Tables

**Table 1:** Main clinical characteristics in people with idiopathic Parkinson's disease (*n* = 456).

Items	Variables
Age, years^a^	60.7 ± 11.3
Cognition status (Mini-Mental Status Examination)^a^	26.2 ± 11.7
Sex^b^	
Male	277 (60.7%)
Female	179 (39.3%)
Illness duration, years^a^	5.5 ± 5.6
HY stage 1^b^	212 (46.4%)
HY stage 2^b^	167 (36.6%)
HY stage 3^b^	34 (7.9%)
HY stage 4^b^	43 (9.00%)
Levodopa equivalent daily dose^a^	766.50 ± 285.15

^a^Mean ± SD. ^b^Number (%).

**Table 2:** Factor analysis for PDSS-2 in people with idiopathic Parkinson's disease (*n* = 456).

Items	Factor 1 (PD motor symptoms at night)	Factor 2 (PD symptoms at night)	Factor 3 (sleep disturbances)
Bad sleep quality	—	—	0.87
Difficulties falling asleep	—	—	0.70
Difficulties staying asleep	—	—	0.76
Restlessness of legs or arms at nights	0.85	—	—
Urge to move your legs or arms	0.84	—	—
Distressing dreams at night	—	0.66	—
Distressing hallucinations at night	—	0.68	—
Get up at night to pass urine	0.64	—	—
Uncomfortable and immobility at night	0.66	—	—
Pain in arms or legs	0.82	—	—
Muscle cramps in your arms or legs	0.83	—	—
Painful posturing in the morning	0.73	—	—
Tremor on waking	0.47	—	—
Tired and sleepy after waking in the morning	0.62	—	—
Snoring or difficulties in breathing	—	0.76	—

**Table 3:** Interitem correlation for PDSS-2 in people with idiopathic Parkinson's disease (*n* = 456).

Items	1	2	3	4	5	6	7	8	9	10	11	12	13	14	15
1	1														
2	0.43	1													
3	0.55	0.68	1												
4	0.22	0.51	0.55	1											
5	0.24	0.56	0.57	0.91	1										
6	0.22	0.39	0.50	0.54	0.58	1									
7	0.21	0.40	0.44	0.61	0.63	0.70	1								
8	0.21	0.26	0.33	0.44	0.43	0.36	0.49	1							
9	0.21	0.47	0.46	0.62	0.63	0.51	0.52	0.44	1						
10	0.23	0.51	0.55	0.83	0.82	0.58	0.66	0.49	0.68	1					
11	0.22	0.44	0.53	0.78	0.80	0.55	0.59	0.46	0.66	0.84	1				
12	0.27	0.44	0.51	0.67	0.70	0.53	0.58	0.41	0.61	0.73	0.73	1			
13	0.21	0.47	0.44	0.53	0.55	0.42	0.48	0.41	0.49	0.55	0.51	0.56	1		
14	0.27	0.45	0.44	0.60	0.60	0.42	0.49	0.41	0.50	0.56	0.56	0.61	0.55	1	
15	0.21	0.24	0.29	0.41	0.43	0.42	0.44	0.37	0.39	0.46	0.44	0.44	0.40	0.31	1

1, bad sleep quality; 2, difficulties falling asleep; 3, difficulties staying asleep; 4, restlessness of legs or arms at nights; 5, urge to move your legs or arms; 6, distressing dreams at night; 7, distressing hallucinations at night; 8, get up at night to pass urine; 9, uncomfortable and immobility at night; 10, pain in arms or legs; 11, muscle cramps in your arms or legs; 12, painful posturing in the morning; 13, tremor on waking; 14, tired and sleepy after waking in the morning; 15, snoring or difficulties in breathing.

## Data Availability

The data used to support the findings of this study are included within the article.
